# A Highly Sensitive and Specific Probe-Based Real-Time PCR for the Detection of *Avibacterium paragallinarum* in Clinical Samples From Poultry

**DOI:** 10.3389/fvets.2021.609126

**Published:** 2021-04-12

**Authors:** Suresh V. Kuchipudi, Michele Yon, Meera Surendran Nair, Maurice Byukusenge, Rhiannon M. Barry, Ruth H. Nissly, Jen Williams, Traci Pierre, Tammy Mathews, Eva Walner-Pendleton, Patricia Dunn, Denise Barnhart, Sean Loughrey, Sherrill Davison, Dona J. Kelly, Deepanker Tewari, Bhushan M. Jayarao

**Affiliations:** ^1^Animal Diagnostic Laboratory, Pennsylvania State University, University Park, PA, United States; ^2^Center for Infectious Disease Dynamics, Pennsylvania State University, University Park, PA, United States; ^3^Pennsylvania Animal Diagnostic Laboratory, New Bolton Center, University of Pennsylvania, Philadelphia, PA, United States; ^4^Pennsylvania Veterinary Laboratory, Harrisburg, PA, United States

**Keywords:** infectious coryza, respiratory disease, recN gene, *A. paragallinarum*, real-time PCR

## Abstract

*Avibacterium paragallinarum* (historically called *Hemophilus paragallinarum*) causes infectious coryza (IC), which is an acute respiratory disease of chickens. Recently, outbreaks of IC have been reported in Pennsylvania (PA) in broilers, layer pullets, and laying hens, causing significant respiratory disease and production losses. A tentative diagnosis of IC can be made based on history, clinical signs, and characteristic gross lesions. However, isolation and identification of the organism are required for a definitive diagnosis. Major challenges with the bacteriological diagnosis of *A. paragallinarum* include that the organism is difficult to isolate, slow-growing, and can only be successfully isolated during the acute stage of infection and secondary bacterial infections are also common. As there were very limited whole genomes of *A. paragallinarum* in the public databases, we carried out whole-genome sequencing (WGS) of PA isolates and based on the WGS data analysis; we designed a novel probe-based PCR assay targeting a highly conserved sequence in the *recN*, the DNA repair protein gene of *A. paragallinarum*. The assay includes an internal control, with a limit of detection (LOD) of 3.93 genomic copies. The PCR efficiency ranged between 90 and 97%, and diagnostic sensitivity of 98.5% compared with conventional gel-based PCR. The test was highly specific, and no cross-reactivity was observed with other species of *Avibacterium* and a range of other common poultry respiratory viral and bacterial pathogens. Real-time PCR testing on 419 clinical samples from suspected flocks yielded 94 positives and 365 negatives in agreement with diagnostic bacterial culture-based detection. We also compared the *recN* PCR assay with a previous *HPG-2* based real-time PCR assay which showed a PCR efficiency of 79%.

## Introduction

Infectious coryza (IC) is an acute upper respiratory disease of growing broilers and layers, caused by *Avibacterium paragallinarum*, a gram-negative bacterium previously called *Haemophilus paragallinarum* (1, 2). The illness is associated with reduced egg production in layers and decreased bodyweight due to impaired food and water consumption in broilers (1). The most common clinical signs in chickens infected with *A. paragallinarum* include facial edema, nasal exudates, sneezing, and conjunctivitis (3). Previously, Page classified the bacterium with a slide agglutination test into serovars A, B, and C (4) whereas the modified Kume scheme based on hemagglutination test and its modifications describe 9 serovars within the species (5–7).

A tentative diagnosis of IC is often made based on history, clinical signs, and characteristic gross lesions. Isolation and identification of the organism are mostly hindered by its fastidious growth characteristics as well as the concurrent colonization of other bacteria in the same respiratory niches. Furthermore, the chronic stage of infection, prior antimicrobial treatments and any delay in sample processing have been shown to interfere with effective recovery of *A. paragallinarum* from diagnostic samples using conventional bacteriological methods (1). Despite the worldwide distribution of *A. paragallinarum* and a major cause of significant economic losses to the poultry industry; the true prevalence, incidence, and overall disease dynamics of IC in poultry flocks is not well-understood. The lack of rapid and sensitive diagnostic tools is one of the reasons for the limited understanding of the ecology and epidemiology of IC.

Recently, IC outbreaks have been reported from multiple states in the US and IC is now considered as an emerging respiratory disease of chickens in the North Eastern US. Since early 2019, several outbreaks of IC have been reported in broilers, layer pullets and laying hens, in Pennsylvania (PA) causing high morbidity with significant respiratory illness and significant production losses (8). IC continues to be reported from adjacent states like Delaware and Maryland and remains a major poultry health concern. This necessitates the development of advanced real-time PCR assays employing amplicon-specific probes, which are highly sensitive and precise for the rapid and accurate detection of pathogens from clinical samples.

## Materials and Methods

### Real-Time PCR Assay Design

Based on the comparative genome analysis of different PA isolates of *A. paragallinarum* that we generated using Illumina MiniSeq platform (Genbank accession #CP051642, CP051641, CP051640, CP051639, CP051638, CP051637, and CP051636) and several other sequences in GenBank, we determined that the DNA repair protein gene *recN* was a highly conserved gene. Previous studies also confirmed that *recN* gene is present in all serovars of *A. paragallinarum* and was considered as a potential housekeeping gene for gene expression studies (9). We designed specific primers and probe targeting a 99bp region of the *recN* gene of *A. paragallinarum* using Primer Express 3.0.1 software (Applied Biosystems, Foster City, CA).

### Synthetic Gene Fragments to Establish Limit of Detection

Two hundred and forty four base pair (bp) synthetic double-stranded DNA fragment for *recN* gene amplicon (gBlock Gene Fragment- Integrated DNA Technologies, Coralville, IA) was used as a positive standard to determine the limit of detection (LOD) for the assay. The number of genomic copies per μL was determined using the molecular weight of the gBlock as per the manufacturer's instructions. Ten-fold serial dilutions of the standard were made starting from 10^3^ to generate a standard curve using three technical replicates for each dilution. The synthetic DNA fragments are used to avoid the problem of copy number variation in different strains and a standardized dilution series could be thus used instead of the reference strain standards.

### Bacteria and Viruses Used for Assessment of Cross-Reactivity

Reference strain of *A. paragallinarum* (ATCC 29545) along with 37 clinical isolates from commercial flocks of PA, cultured and identified as *A. paragallinarum* in the laboratory were used to evaluate the diagnostic sensitivity of the developed assay. The clinical *A. paragallinarum* isolates were identified using biochemical reactions, growth conditions, matrix- assisted laser desorption/ionization time-of-flight identification system (MALDI-TOF MS, Bruker Daltonics, Bremen, Germany) and a positive result in a conventional PCR specific for *A. paragallinarum* developed by Chen et al. (10). Analytical specificity was tested using various *Avibacterium* sp. isolates including *A. avium* (ATCC 29546), *A. gallinarum* (ATCC 13360), *A. gallinarum* (ATCC 13361), *A. volantium* (ATCC 14385), and common respiratory pathogens including infectious bursal disease virus (IBDV D-78 strain), infectious bronchitis virus (IBV- Georgia 2008 type strain), infectious laryngotracheitis virus (ILTV—Lt-IVAX vaccine strain), fowlpox virus, Newcastle disease virus (NDV- Lasota strain), avian influenza virus (AIV), avian reovirus (S-1133 strain)*, Mycoplasma gallisepticum* (6/85 strain), and *Pasteurella multocida* (M-9 strain). All bacterial and virus isolates were obtained from either the American Type Culture Collection (ATCC) or from the National Veterinary Services Laboratory (NVSL), Ames, Iowa, USA.

### Culture Conditions and Genomic DNA Preparation

*Avibacterium* sp. were cultured on chocolate agar (CHOC; Remel, ThermoFisher Scientific, Waltham, MA) for 48 h at 37°C with 5–7% CO_2_. DNA was extracted from plates with purified colonies using MagMAX Pathogen RNA/ DNA Kit (Applied Biosystems).

### Conventional PCR

Conventional PCR was performed on 2 μL of extracted DNA to amplify a 500 bp region as previously described (10). Briefly, 3 μL each of forward (5′-CAA GGT ATC GAT CGT CTC TCT ACT −3′) and reverse (5′-TGA GGG TAG TCT TGC ACG CGA AT-3′) primers were used at 10 μM concentrations in a total reaction volume of 50 μL containing AmpliTaq Gold® DNA Polymerase (Applied Biosystems) and 25 mM magnesium chloride (Promega) in GeneAmp assay buffer II (Applied Biosystems). Reactions were carried out using a 9,600 GeneAmp PCR system (Perkin Elmer, Waltham, MA) with the following conditions: 95°C 4 min; 40 cycles of 94°C 1 min, 63°C 1 min, 72°C 30 s, and a cycle of 94°C 1 min, 63°C 1 min, 72°C 10 min. The reaction product (20 μL) was visualized on a 1.5% agarose gel containing 0.5 mg ethidium bromide per mL of agarose solution.

### Real-Time PCR

Purified primers and probe were purchased from Integrated DNA Technologies (IDT). The PCR reaction was prepared in a volume of 25 μl consisting of 5 μL of the template DNA, 12.5 μL of master mix solution (VetMAX-Plus qPCR Master; Applied Biosystems), 1 μL of each 10 μM forward (*recN* FWD Primer 5′- GAACAAGACCCTTATCGCTTACAAG −3′) and reverse (*recN* REV Primer 5′- ACTCACTAATTCTTCCGCTTTTACATT −3′) primers, 0.3 μL of 10 μM fluorogenic probe [*recN* Probe 5′ [FAM] CAGGCACTGCAATTAGCCCGCAA [BHQ-1]-3′], 1 μL of Xeno IPC/VIC) and 4.2 μL of DNase and RNase-free water. The reactions were performed using a 7,500 Fast Real-Time PCR System (Applied Biosystems) with the following cycling conditions: an enzyme activation cycle at 95°C for 10 min, followed by 40 cycles of 95°C for 15 s and 60°C for 45 sec. All samples were run in triplicates, and the experiment was repeated thrice using nuclease free water as no target control in all reactions. With an aim to ensure accurate PCR results and to reduce the likelihood of false negatives VetMAX Xeno IPC DNA (Applied Biosystems) in a concentration of 10,000 copies/μL was introduced at the nucleic acid isolation/preparation step and carried through the qPCR workflow as an internal control.

### Analytical Sensitivity-Limit of Detection

Serial 10-fold dilutions of a gBlock gene fragment of *recN* gene of *A. paragallinarum* was PCR tested in triplicates, and cycle threshold (Ct) values for each were plotted to obtain standard curves, slopes, and R^2^ values. The gBlock was resuspended to 10 ng/ml stock solution with approximately 1.52 × 10^12^ copies/ml. The LOD for *A. paragallinarum* PCR assay was estimated from the values of the standard curve generated through the assay. The assay included three technical replicates.

### Analytical Specificity of recN PCR Assay

Pathogens most frequently encountered in clinical samples from chickens with respiratory illnesses were tested on this real-time assay to ensure specificity. The genomic content extraction from the pathogens were performed using the MagMAX Pathogen RNA/DNA kit as described previously.

### Evaluation of Diagnostic Sensitivity and Specificity

#### Identification of Clinical Isolates

Thirty-seven clinical isolates from PA commercial flocks cultured and identified as *A. paragallinarum* using bacterial culture in the Pennsylvania State University Animal Diagnostic Laboratory were used. DNA was extracted from plates with purified colonies using the extraction kit, MagMAX Pathogen RNA/ DNA kit (Applied Biosystems). Although the colony count was not standardized between extraction preparations, the initial DNA concentrations yielded comparable Ct values. The isolates were also tested using the conventional PCR described above.

#### Direct Detection in Clinical Samples

Clinical samples (*n* = 419) comprising swabs from sinus, choana, oropharynx, lung, air sac or trachea from chickens with suspected respiratory disease submitted to the Pennsylvania State University Animal Diagnostic Laboratory (ADL) were used for evaluation of diagnostic sensitivity. Swabs were vortexed and agitated well in saline and 300 μl of the broth was used to extract DNA using the MagMAX Pathogen RNA/ DNA kit (Applied Biosystems) following the manufacturer's instructions. The samples were also simultaneously cultured for bacterial isolation and identification for comparison.

### Comparative Efficacy of the New recN qPCR vs. Previous HPG-2 qPCR

The efficiency of *recN* based PCR was compared to a previously validated assay targeting the *HPG-2* (*Haemophilus paragallinarum*, the historical name for *A. paragallinarum*) region of the bacterium (11). DNA was extracted from broth cultures of *A. paragallinarum* (ATCC 29545) using the MagMAX Pathogen RNA/ DNA Kit. The DNA extract from each 10-fold dilution was PCR tested in triplicate, and cycle threshold (Ct) values were plotted to obtain standard curves, slopes, and R^2^ values.

As per the published protocol, *HPG-2* PCR reaction was prepared in a volume of 25 μl consisting of 2 μL of the template DNA, 12.5 μL of master mix solution (VetMAX-Plus qPCR Master; Applied Biosystems), 1.76 μL of each 10 μM forward (HPG-2 FWD Primer 5′- GCAAAAGACTACCAGCAAGGATAAT −3′) and reverse (HPG-2 REV Primer 5′- CCTTACCCAAATATAATGTTCCACATT −3′) primers, 0.66 μL of 10 μM fluorogenic probe (Probe 5′ 6FAM-TCCTAGTTAG- CATTATTGC-MGBNFQ 3′), 1 μL of Xeno IPC/VIC) and 2.32 μL of DNase and RNase-free water. The reactions were carried out on Applied Biosystems 7,500 Fast Real time PCR machine with cycling parameters as: 50°C for 2 min, 95°C for 10 min, 40 cycles of 95°C for 15 s, followed by 60°C for 1 min.

### Statistical Analysis

Cohen's kappa was estimated to determine the agreement between the two tests. The level of statistical significance was set at 0.05. The sensitivity and specificity of the *recN* based PCR were calculated by comparing it with the conventional gel-based assay.

## Results

### Analytical Sensitivity and Specificity

**Figure 1:**
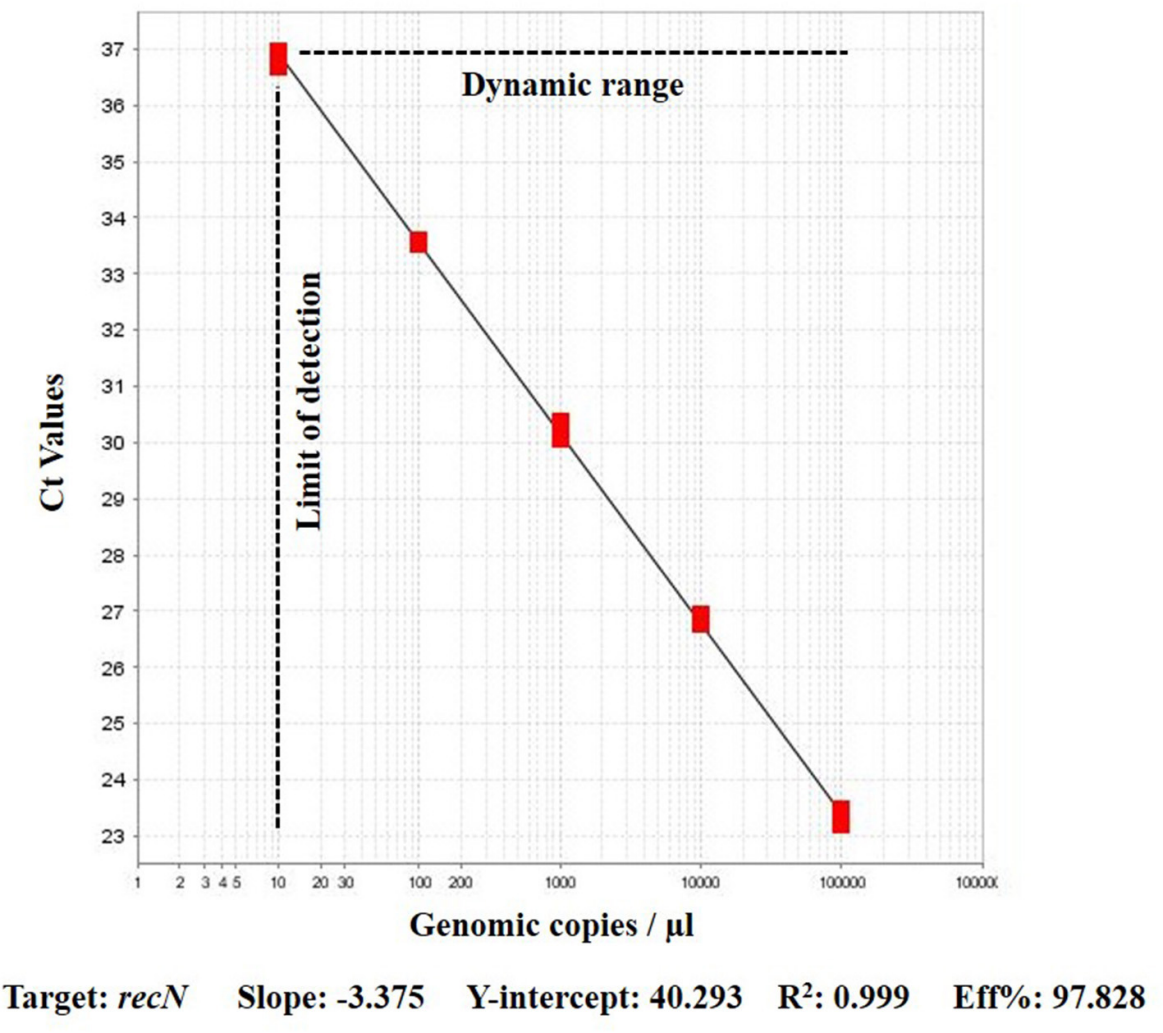
Performance of real-time PCR assay on serial dilutions of gBlock genomic fragments for *A. paragallinarum* recN target.

The efficiency of *recN* based assay using genomic fragments of gBlock dilutions was between 90 and 97% with R^2^ 0.99. The 3.93 copy numbers samples produced a mean Ct of 35.97, which resulted in a consistent LOD for the assay (Figure 1).

Using single-tube extraction for *A. paragallinarum* ATCC 29545 with Xeno DNA on serial dilutions, yielded positive detection on conventional endpoint gel-based PCR up to 10^−4^ dilutions. However, the *recN* based real-time PCR detected *A. paragallinarum* ATCC 29545 DNA in all dilutions up to 10^−6^ dilution. No cross reactivity was found with other tested respiratory pathogens (Ct value > 36; Table 1).

**Table 1:** Analytical specificity and sensitivity of recN based PCR.

**Sample**	**Target**	**Ct Value**
*Avibacterium paragallinarum*	*recN*	19.574
*Avibacterium avium*	*recN*	Undetrmined
*Avibacterium volantium*	*recN*	Undetrmined
*Avibacterium gallinarum*	*recN*	Undetrmined
*Pasteurella multocida*	*recN*	Undetrmined
*Mycoplasma gallisepticum*	*recN*	Undetrmined
*Ornithobacterium rhinotracheale*	*recN*	Undetrmined
*Gallibacterium anatis*	*recN*	Undetrmined
*Escherichia coli*	*recN*	Undetrmined
*Staphylococcus aureus*	*recN*	Undetrmined
Avian influenza virus	*recN*	Undetrmined
Avian reovirus	*recN*	Undetrmined
Infectious bursal disease virus (IBDV)	*recN*	Undetrmined
Infectious bronchitis virus (IBV)	*recN*	Undetrmined
Infectious laryngotracheitis virus (ILTV)	*recN*	Undetrmined
Fowlpox virus	*recN*	Undetrmined
Newcastle disease virus (NDV)	*recN*	Undetrmined

### Diagnostic Sensitivity and Specificity

Overall agreement between the real time *recN* based PCR and conventional PCR testing performed at the laboratory using the *A. paragallinarum* isolates was 98.5%, including 36 of 37 real time PCR-positive and 30 of 30 negative cultures producing matching results. The Ct values for positive cultures ranged between 23 and 36. The internal positive control (XENO—IPC) from those isolated using the MagMAX kit amplified with an average Ct of 30.82.

Real-time PCR testing on 419 clinical samples from suspected flocks yielded 100% (94 positives and 365 negatives) agreement with diagnostic bacterial culture followed by identification by MALDI-TOF analyzer.

### Comparison PCR

**Figure 2:**
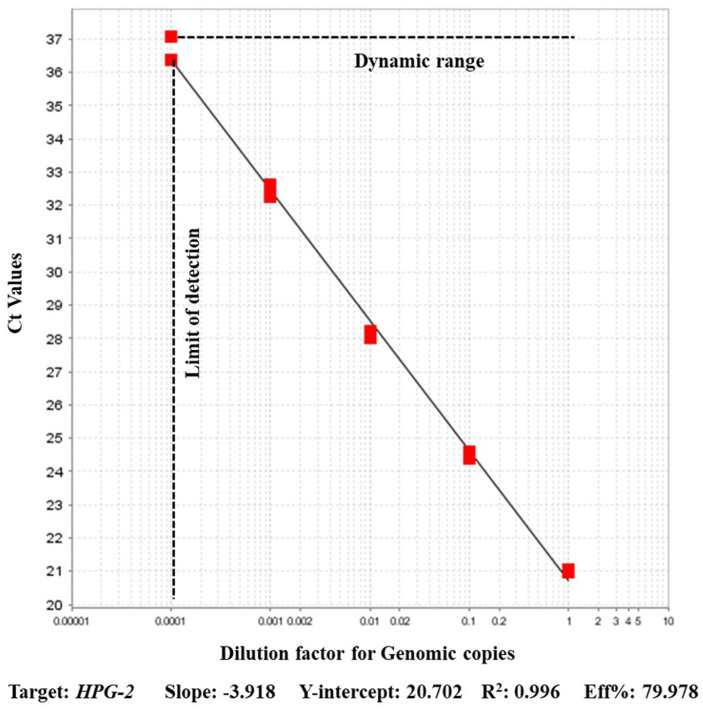
Performance of real-time PCR assay on serial dilutions of *Avibacterium paragallinarum* (ATCC 29545) for the detection of *HPG-2* target.

A strong agreement was found among the *recN* based assay results and the previously validated *HPG-2* based PCR. The *HPG-2* based PCR detected *A. paragallinarum* ATCC 29545 DNA in dilutions up to the 10^−4^ dilution. The efficiency of *HPG-2* based assay evaluated from the standard curve generated using the dilutions was 79% with R^2^ 0.996 (Figure 2). The positive amplification control included in the assay amplified with a Ct value of 16.614 in *recN* based assay whereas that using *HPG-2* as the target had the amplification only at a Ct of 18.6415 (Table 2). A strong agreement was found between the outcomes of the new test and *HPG-2* PCR (kappa = 0.96; *p* < 0.05).

**Table 2:** Ct values for recN and HPG-2 targeted PCRs.

**Sample**	**Target**	**Ct Value**
*Avibacterium paragallinarum* (ATCC 29545)	recN	16.61224
	HPG-2	18.6415

## Discussion

Infectious coryza continues to be a global threat to the poultry industry and has recently been recognized as an emerging infectious disease of poultry in the eastern US. *A. paragallinarum* bacterial respiratory disease of poultry, which requires robust methods for accurate diagnosis. Bacteriological diagnosis of *A. paragallinarum* is often challenging due to its fastidious nature. It is well-known that real-time PCR (rtPCR) assays employing amplicon-specific probes are highly sensitive and precise for the rapid and accurate detection of pathogens from clinical samples (12–14).

Previously, a gel-based PCR assay published in 1996 has been extensively used for the detection of *A. paragallinarum* from diagnostic samples (10). Based on this *HPG* gel based PCR assay, a 5′ Taq nuclease assay targeting a smaller region of the gene called *HPG-2* of *A. paragallinarum* was developed in 2008 (15) and the workflow was recently validated in 2019 (11). However, these assays are designed based on the study published in 1996, when the whole genome sequence information of *Avibacterium paragallinarum* was not available. Furthermore, recently a lateral flow test has been developed for the rapid detection of *A. paragallinarum* (16). While this assay offered a rapid diagnosis, it suffered from lack of specificity and did not offer ability to distinguish the commensal species of *Avibacterium* such as *A. avium, A. endocarditis, A. gallinarum, and A. volantium*.

We generated whole genome sequences (WGS) of 18 *A. paragallinarum* isolates from recent outbreaks in Pennsylvania (January through April 2019). Based on the analysis of WGS of PA isolates and the ATCC reference strain, we developed a novel probe based real-time PCR assay. The *recN* gene in *A. paragallinarum* is a DNA-dependent repair protein (17). Previous reports on *recN* based phylogenetic comparison showed a very narrow species relationship between strains of *A. volantium, A. avium, A. endocarditidis*, and other *Avibacterium* species (17). A high *recN* gene divergence was also reported between the *A. paragallinarum* strains and groups of other isolates (17). To predict the whole-genome similarity, G-C content and phylogeny of selected taxa within the *Pasteurellaceae, recN* was also used as a candidate gene for multi-locus sequencing (18). Furthermore, our whole genome sequence analysis of PA isolates and other sequences in GenBank, identified *recN* as the highly conserved gene across all the three serovars of *A. paragallinarum*. Therefore, the newly developed *recN* PCR is an invaluable tool to diagnose low levels of *A. paragallinarum* rapidly and accurately from clinical samples. To date 419 clinical samples from suspected flocks were tested using the *recN* based PCR yielded 100% agreement with diagnostic bacterial culture-based detection.

In this study a strong agreement was found among the *recN* based assay results and the HPG-2 based PCR. Although, the previous study reported 89–111% efficiency with HPG-2 detection (11), we were only able to replicate the HPG-2 based assay with 79% efficiency. The new *recN* based qPCR assay has consistently been performed with a higher efficiency of 90–97%. PCR efficiency is a significant factor for the quantification of the target DNA in unknown samples (13). Consequently, PCR efficiency affects the detection and quantification limits of a PCR assay. Clinical samples for bacteriological diagnosis can have varying levels of the bacterial load depending on the type of sample, collection method, and stage of the disease. Sequence-verified, dsDNA gBlocks Gene Fragments, used to generate standard curve in this study are a great alternative to culture -based methods as it reduces the chances for pipetting error, and saves time diluting and plating multiple bacterial isolates.

## Data Availability Statement

The datasets presented in this study can be found in online repositories. The names of the repository/repositories and accession number(s) can be found in the article/supplementary material.

## Author Contributions

SK conceived and designed the study. MB and SK analyzed the WGS data and designed the PCR assay. MY, JW, RB, and RN carried out the PCR assay development and validation. PD, EW-P, SD, DK, and SL carried out the necropsies and collection of clinical samples. TP, TM, and DB carried out the bacterial isolation and identification. DT, BJ, SK, MS, and RN analyzed the data and interpretation. MS, MY, and SK drafted the manuscript. All authors reviewed and approved the final version of the manuscript.

## Conflict of Interest

The authors declare that the research was conducted in the absence of any commercial or financial relationships that could be construed as a potential conflict of interest.
